# Spaced training enhances equine learning performance

**DOI:** 10.1007/s10071-021-01580-7

**Published:** 2021-12-03

**Authors:** Frederick R. Holcomb, Kristi S. Multhaup, Savannah R. Erwin, Sarah E. Daniels

**Affiliations:** 1 Psychology Department, Davidson College, Davidson, NC 28035 USA; 2 Present Address: Veterinary Medicine, Texas A&M, College Station, TX USA; 3 Present Address: Psychology & Neuroscience, Duke University, Durham, NC USA; 4 Present Address: Savannah, GA USA

**Keywords:** Spaced and massed learning, Horse behavior, Equine cognition, Training schedule

## Abstract

This field experiment examined whether the well-documented benefit of spaced over massed training for humans and other animals generalizes to horses. Twenty-nine randomly selected horses (*Equus ferus caballus*) repeatedly encountered a novel obstacle-crossing task while under saddle. Horses were randomly assigned to the spaced-training condition (2 min work, 2 min rest, 2 min work, 2 min rest) or the massed-training condition (4 min work, 4 min rest). Total training time per session and total rest per session were held constant. Days between sessions (*M* = 3) were held as consistent as possible given the constraints of conducting research on a working ranch and safety–threatening weather conditions. During each training session, the same hypothesis-naïve rider shaped horses to cross a novel obstacle. Fifteen of 16 horses in the spaced-training condition reached performance criterion (94% success) while only 5 of 13 horses in the massed-training condition reached performance criterion (39% success). Horses in the spaced-training condition also initiated their first obstacle-crossing faster than horses in the massed-training condition and were faster at completing eight crossings than horses in the massed-training condition. Overall, task acquisition was higher for horses undergoing spaced training despite both groups experiencing the same total work and rest time per session. These findings generalize the learning-performance benefit observed in human spaced practice to horses and offer applied benefit to equine training.

## Introduction

Equine training regimens aim to effectively convey a job to an animal whose evolutionary priority is to avoid situations that increase its likelihood of being preyed upon (Brubaker and Udell 2016; Christensen et al. 2011). While equine training can take many forms with divergent goals, the common thread is shaping a desired behavior in place of a more natural fight-or-flight response (Christensen et al. 2010). Much of the behavioral research that has been done with equids has focused on social learning (Krueger et al. 2014; Rørvang et al. 2015), cognition and perception (Gabor and Gerken 2010; Hanggi 2005; Matsuzawa 2017; Osthaus et al. 2013; for a review see Brubaker and Udell 2016), and reinforcement (Leblanc and Duncan 2007). Equine cognition studies, however, rarely have been conducted in typical, dynamic training environments (Cooper 2007; McGreevy and McLean 2010). This is a known issue with academic research, and, on the flip side, scholars have lamented the dearth of learning theory knowledge among most equine practitioners (Creighton 2007; Ladewig 2007; McCall 2007). The present research was conducted on a working ranch and used sound experimental design (e.g., random selection, random assignment to condition), demonstrating that studies can contribute to a theoretical perspective while simultaneously providing data to facilitate applied horse–human interactions (Goodwin 2007; Randle 2016).

The present field study aims to inform equine training practices. It utilized the combination of a ridden horse (*Equus ferus caballus*) and a novel obstacle-crossing task to evaluate whether best practices in human learning are replicated in and extended to horses. Specifically, a robust strategy for improving human learning is the *spacing effect*: recall of material is better after studying is interspersed with breaks than when the same amount of studying happens over an uninterrupted period (Cepeda et al. 2006; Dunlosky et al. 2013; Kornell et al. 2010). The benefit of spaced practice for human learning performance has been observed both at the level of a single training session (*intra-session spacing*) and between sessions (*inter-session spacing*) (Crowder 1976; Kornell et al. 2010).

The sparse literature on the spacing effect in nonhuman animal species suggests the effect generalizes to rats (Bello-Medina et al. 2013), mice (Aziz et al. 2014), dogs (Demant et al. 2011), and pigs (Karas et al. 1962). Indeed, this pattern has also been shown by honeybees (Menzel et al. 2001) and drosophila (San Martin et al. 2017). Given this training phenomenon generalizes across species, we hypothesize that horses will show a benefit of spaced training compared with massed training. Importantly, exploring the spacing effect in horses offers the possibility of applied as well as theoretical benefits. Any training schedule that increases efficiency of training can reduce stress and potential injury for both the equine subject and the human trainer (Murphy and Arkins 2007). Unfortunately, the current evidence regarding the spacing effect in *Equus ferus caballus* is inconclusive. An early study that cued ponies to clear a hurdle or back up to avoid a shock found that increasing spacing between training sessions (inter-session spacing) by decreasing the number of sessions per week improved the acquisition efficiency of learned avoidance (Rubin et al. 1980). In other words, the ponies showed the spacing effect seen in other animals. By contrast, teaching yearling horses to be driven and ridden using a training schedule that included days off (increasing inter-session spacing) was *less* effective than daily training (Kusunose and Yamanobe 2002). Surprisingly, this is the opposite of a spacing effect and might have been a product of subject age (immature vs mature horses), task complexity (applied training task vs simplified conditioning task), or trainer variability (multiple trainers utilized). These discrepant findings point to the need for additional research about the spacing effect in horses. The present research uses the high internal control of Rubin et al. (1980) and the high external generalizability of Kusunose and Yamanobe (2002). Given that equine training schedules are often affected by circumstances beyond the trainer’s control, the present field experiment moved away from inter-session spacing and examined whether *intra-session* spacing can improve equine learning performance.

This field experiment’s examination of intra-session spacing on equine learning performance (a) extends the spacing effect literature into an understudied species, (b) provides data on a component of the spacing effect that has not yet been studied in horses, namely the effects of *intra-session* spacing, (c) employs an experimental manipulation that generalizes to multiple facets of applied equine training and riding, and (d) extends the study of the spacing effect into contexts that use negative reinforcement (cf. Rubin et al. 1980). The primary hypothesis was that compared with massed training, spaced training will yield a higher percentage of horses meeting the performance criterion.

## Materials and methods

### Sample

A mixed herd (quarter, paint, and crosses) of 46 horses from the HF Bar Ranch in Saddlestring, Wyoming, USA, was used in pilot testing and formal data collection. All subjects were used as guest horses for ranch trail riding (maximum of two trail rides per day at a leisurely pace) and they experienced the same nightly turnout schedule with ad libitum access to grass and water. Subjects were randomly selected from HF Bar’s approximately 200-horse herd, which is housed in rotating pastures on the 9000-acre ranch. Detailed information on individual age and breeding was unavailable for each horse but all subjects were typical adult ranch horses ranging roughly from 8 to 16 years old. Forty-five were geldings and one was a mare. Nine horses were used in pilot testing to establish task difficulty. In the main experiment, eight horses (five in the spaced-training condition and three in the massed-training condition) reached performance criterion in the first session and were excluded from further data collection because the task was deemed to be too easy for those individuals (ceiling performance). Prior to data collection, this study was approved by the Davidson College Institutional Animal Care and Use Committee (Protocol # 6-14-01) as well as the owners of the HF Bar Ranch.

### Novel task

**Fig. 1:**
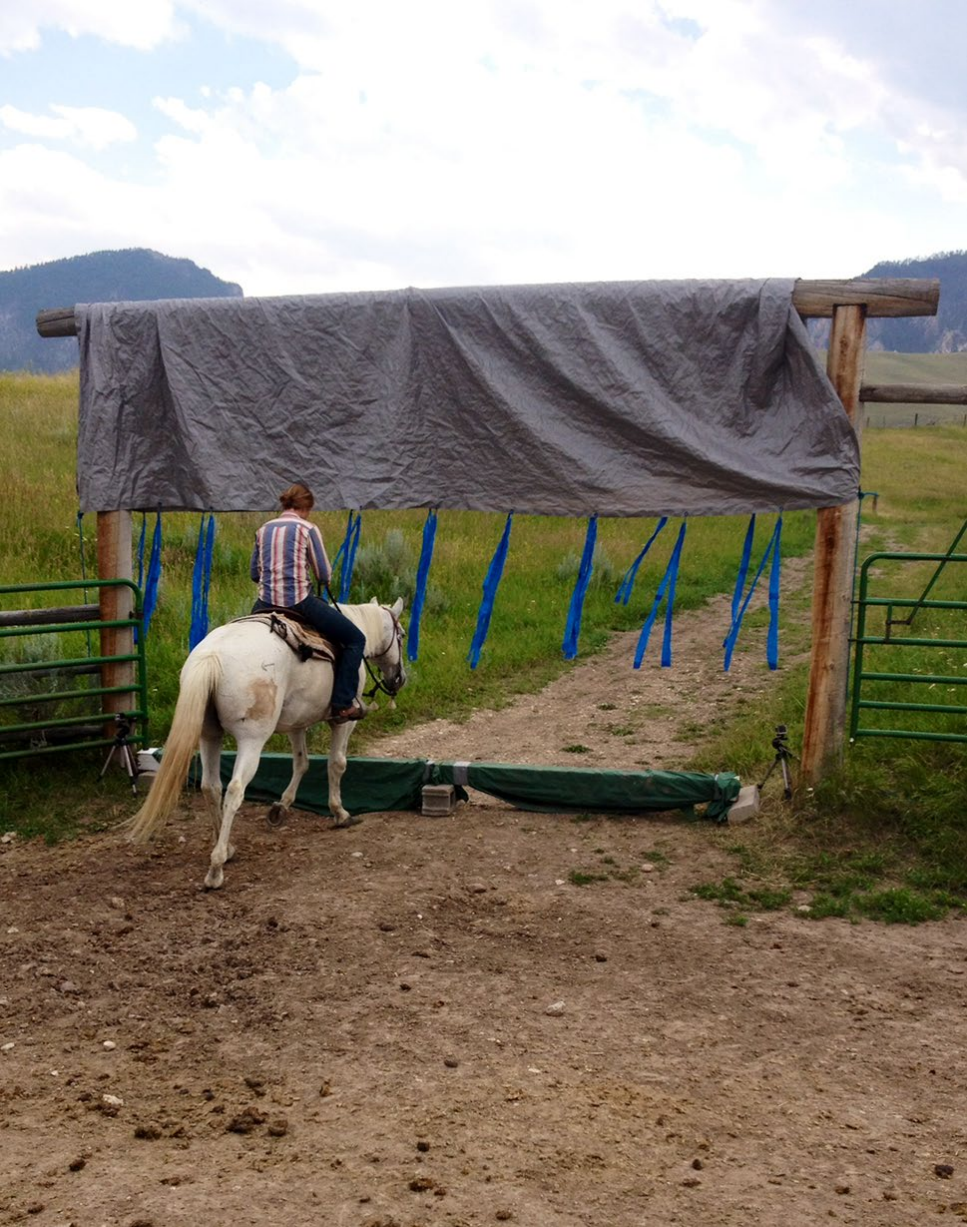
The novel obstacle-crossing task. The experimental obstacle including ground component that needed to be stepped over as well as a hanging tarp that moved freely above the horses as they crossed. The horse with hypothesis-naïve rider is in the process of one crossing on the task where learning criterion for success was completing eight crossings in a single session

As prey animals, horses are often frightened by novel stimuli making habituation an important part of equine training (Christensen et al. 2010, 2011). We conducted pilot work to develop an obstacle and training session time periods that minimized floor and ceiling effects. Pilot horses were not included in the study sample, but they were from the same population. The training task involved carrying a rider fully (all four of the horse’s feet) across a novel obstacle (Fig. 1). A horse reached performance criterion once it crossed the obstacle eight times within a single training session. This task has elements in common with typical tasks that domestic horses face (e.g., trailer loading, entering a gate), but is unlikely to have been encountered by the horses prior to the field experiment.

The obstacle was set up and taken down daily so that horses were exposed to it only during experimental trials. The training location was out of sight and sound of both the corral, where the ranch horses were housed during the day, and turnout pastures, where the horses were housed at night. The dimensions and presentation of the obstacle, and, in turn, the difficulty of the task, were established through initial pilot testing.

### Experimental groups

Horses were randomly assigned to one of two experimental groups: spaced-training or massed-training. Horses in the spaced-training condition (*n* = 16) experienced 2 min of work, 2 min of rest, 2 min of work, and 2 min of rest while horses in the massed-training condition (*n* = 13) experienced 4 min of work, followed by 4 min of rest. The timing allowed us to collect data from a reasonable number of horses in the time that we had available for testing at the working ranch. Importantly, whether in the spaced or massed condition, each horse received the same amount of work (4 min) and rest (4 min) per session. The sample size of 29 horses aligns with comparable studies of equine learning and cognition. Random selection from a large population as well as random assignment and medium-to-large effect sizes support our data interpretation.

### Training

We used as much experimental control as possible in this naturalistic setting to gain the best of both approaches to research (Banaji and Crowder 1989). All training trials were conducted between 1:00 p.m. and 4:00 p.m. while the rest of the herd was held in a separate pasture out of sight and hearing distance from each day’s group of training horses. Horses in both conditions experienced repeated training with the inter-session spacing held as constant as possible on a working ranch (on average, 3 days between sessions). The number of horses run on a given training day varied from 1 to 5; factors such as weather influenced how many horses could be run safely in the training time available. Of the 32 training days, 88% had either equal numbers of horses run in massed and spaced conditions, or the number differed by one across conditions. Toward the end of the training period when no new horses could start the procedure because they would not have the option to train for 5 days, more horses were run in the massed condition than in the spaced condition because they were completing their training. This procedure of training similar numbers of horses in each condition on as many training days as possible, with weather and the like cooperating, reduces possible concerns about the rider reinforcing horses differently early in the study compared to late in the study given the rider’s work contributed to each condition similarly for the vast majority of training sessions.

During training, all subjects were ridden by the same hypothesis-naïve rider (an experienced wrangler at the guest ranch) using a standard O-ring snaffle bit, small-roweled spurs, and a western saddle. Thus, any idiosyncratic rider behaviors contributed to both the spaced and massed training conditions. The rider did not ride the horses during normal work activities on the ranch and had no riding experience with the subjects before the study period. During the work period of each training session, the rider shaped horses to cross the obstacle while the ground researcher recorded training time from a safe distance. The rider used leg (pressure applied to the horse’s side) and rein (pressure applied to the bit) cues to direct the horse’s forward motion toward the obstacle. As is standard in negative reinforcement training, the rider continuously cued the horse while it was hesitating or avoiding the obstacle and ceased cueing while the horse was approaching the obstacle (McLean 2005; McGreevy and McLean 2010). The ground researcher timed the training sessions and directed the rider when to start and stop each work and rest period.

During the rest periods of both training conditions, the horse and rider were led away from the obstacle by a researcher on the ground. The horses were led approximately 15 yards from the obstacle and held by the ground researcher while the rider stayed on the horse. During the rest period the rider ceased all cueing. All training trials were filmed using two GoPro cameras.

### Performance criterion

Pilot testing showed that the ability to complete eight crossings within a single training session indicated task proficiency while minimizing both ceiling and floor effects. Horses that did not complete eight obstacle crossings in a single session after five training sessions were classified as failed to reach performance criterion.

### Video coding

The videos recorded at time of training were tools for further evaluation of task performance. The principal investigator (PI; also the ground researcher at time of training) created 29 separate, horse-specific videos using Adobe Premiere Pro. These videos were reduced to 50% real-time speed to allow for more accurate behavioral coding. Each work session was divided into two 2-min sessions such that hypothesis-naïve video coders could not guess the manipulation or differentiate between experimental conditions. These 2-min segments were then numbered and randomized using an online random number generator so that order of appearance in the compiled video was not consistent with real-time training order.

The PI scored the randomized videos. To check for potential bias of the PI, hypothesis-naïve researchers were recruited for video coding. These coders were unaware of the two training conditions or the hypothesis, and none were present during the data collection. A total of four dependent measures (behaviors) were coded (approach time, speed, spooking, and positionality); each hypothesis-naïve coder scored only one of the behaviors to reduce biases in coding such as halo effects (e.g., Nisbett and Wilson 1977). Hypothesis-naïve coders were trained with a 2-h training session using video from initial pilot testing; a second session was used if coders did not show clear understanding of the behavior.

The primary dependent video measure was how many seconds each horse spent *approaching* the obstacle. The horse was coded as approaching if it initiated any step that would carry it toward the obstacle. Approach time ended if the horse turned away, backed-up, or moved laterally in relation to the obstacle. Two hypothesis-naïve coders scored this measure. Given the continuous nature of this variable, inter-rater reliability was assessed with a correlation between the primary coder and the hypothesis-naïve coders on approach time per session. Inter-rated reliability was assessed with Intra-class Correlation Coefficient (ICC; and accompanying 95% confidence intervals) using SPSS version 26. We report two-way mixed effects models for absolute agreement. The estimated agreement was excellent (Koo and Li 2016) for coder II (ICC = 0.93, 95% CI [0.83, 0.97]; scored 11% of sessions) and moderate for coder III (ICC = 0.74, 95% CI [− 0.14, 0.91]; scored 19% of sessions). The first video scored by coder III shows much lower reliability than subsequent videos; in other words, this coder got better with practice. Exclusion of the first video scored results in excellent inter-rater reliability (ICC = 0.91, 95% CI [0.12, 0.98]), suggesting high reliability for most of the coding. Changes in speed (time spent walking, trotting, or loping) and spooking behavior (horse’s front feet simultaneously suspended above ground) did not yield satisfactory reliability so will not be discussed further. Position in arena required an additional set of videos to be created that had boundary lines superimposed to create four distinct sections. Two coders’ reliability with the primary coder’s times in each arena position all were high (lowest ICC = 0.97, 95% CI [0.95, 0.98]) but the data gathered was redundant with the crossing data initially gathered (when a horse was about to cross it spent more time closer to the obstacle) and did not offer additional insight so will not be discussed further. Given the high inter-rater reliability, the primary coder’s data were used for all approach analyses.

## Results

### Reaching training criterion

**Fig. 2:**
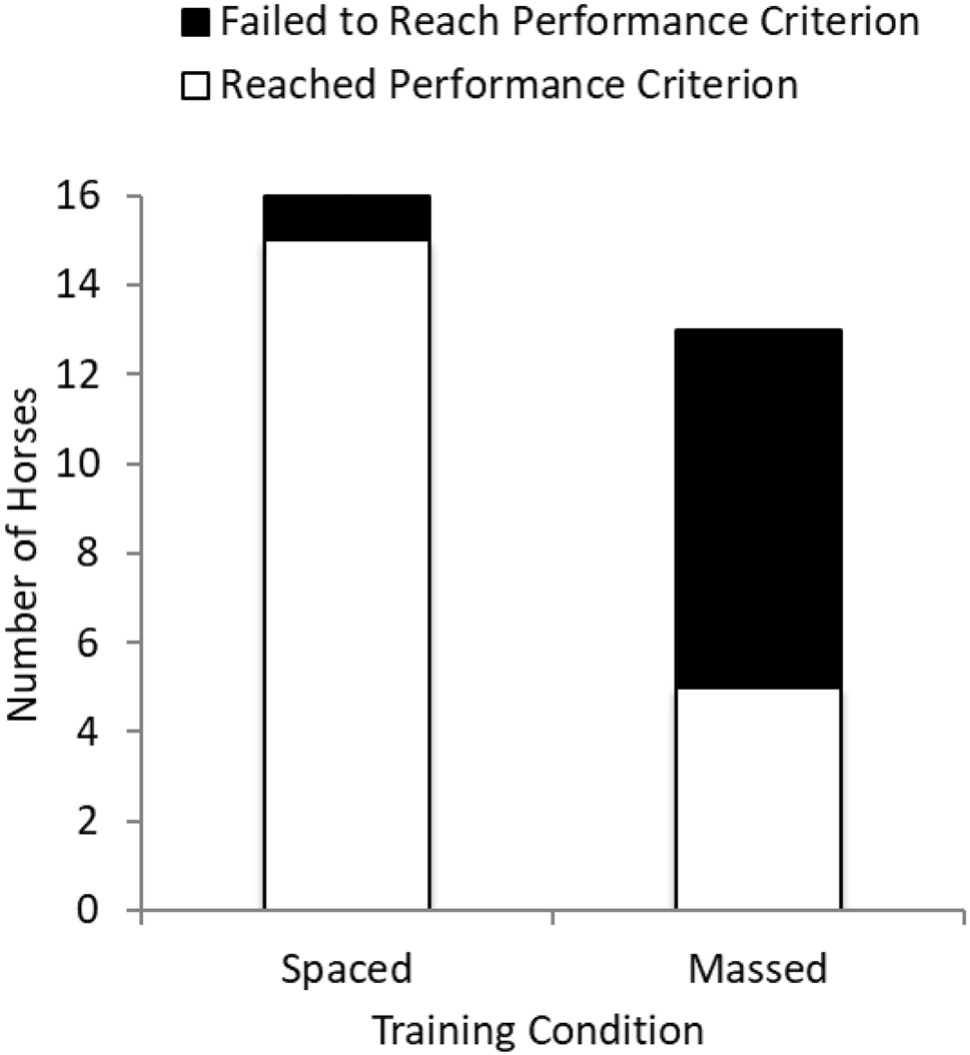
Performance success by training condition. Spaced training resulted in a greater number of horses reaching performance criterion than did massed training

**Fig. 3:**
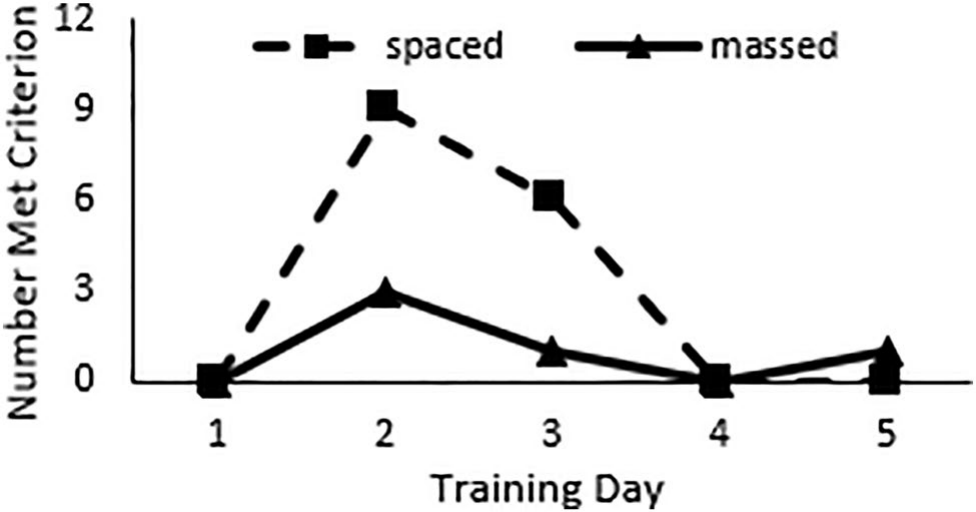
The number of horses that met criterion on each training day. Spaced training resulted in a greater number of horses reaching performance criterion than did massed training

Fifteen out of 16 horses in the spaced-training condition reached performance criterion (94% success) while only 5 of 13 horses in the massed-training condition reached performance criterion (39% success). A chi-square test supported the conclusion that the spaced-training condition led to a higher success rate than the massed-training condition did, *X*^2^ (1, *N* = 29) = 10.24, *p* = 0.001, phi = 0.594, with a large effect size (see Fig. 2). Figure 3 shows the number of horses that met criterion on each training day.

As an additional post hoc exploration of the data, a more lenient performance criterion was also examined: completing at least one obstacle crossing. Again, significantly more horses in the spaced-training condition completed at least one crossing (15 of 16) than did horses in the massed-training condition (8 of 13; 62%), *X*^2^ (1, *N* = 29) = 4.54, *p* = 0.033, phi = 0.395, a medium-to-large effect size.

### Video analyses

To ensure that initial sampling bias did not contribute to the detected differences between conditions, approach behavior in the first 2 min (before the training procedures differed) was compared. Time spent approaching the obstacle during the first 2 min of training was virtually identical for horses in spaced-training (*M* = 1:05 min, *SD* = 0:26) and massed-training (*M* = 1:04 min, *SD* = 0:31) conditions. This finding reduces concerns about sampling bias and supports our interpretation that the distribution of work and rest periods drove the higher rates of reaching criterion performance in the spaced-training group compared with the massed-training group.

Using the coded videos, we examined the time it took the horses who reached criterion (eight crossings in a single session) to start their first crossing and to complete their last crossing. Horses in the spaced-training condition initiated their first crossing faster (*M* = 2:36 min, *SD* = 1:17) than horses in the massed-training condition (*M* = 4:04 min, *SD* = 2:31), *t*(21) = 2.08, *p* = 0.05, Cohen’s *d* = 0.81 (this is the difference in condition means divided by the pooled variance; https://www.socscistatistics.com/effectsize/default3.aspx), a large effect size. No horses completed an obstacle crossing within the first 2 min of training. We also measured the amount of time taken to complete eight crossings (time at which the final foot hit the ground on the horse’s eighth and final crossing) for every horse that reached performance criterion. This analysis should be interpreted with caution given only five horses in the mass condition completed eight crossings, reducing the power of the analysis. Again, horses in the spaced-training condition (*M* = 8:02 min, *SD* = 2:37) were faster at completing eight crossings than horses in the massed-training condition (*M* = 10:15 min, *SD* = 4:47). While this difference did not reach significance, *t*(18) = 1.33, *p* = 0.20, the Cohen’s *d* = 0.57 indicates a medium effect size.

## Discussion

The primary hypothesis was supported: intra-session spaced training led to a significantly higher proportion of horses achieving pre-defined task proficiency than did intra-session massed training. Even when using a more lenient completion criterion, reducing the definition of task proficiency from eight crossings to one crossing, there was still a significant difference between the two training groups that favored intra-trial spaced training. Additionally, of the horses that reached performance criterion, those in the spaced-training condition initiated a first crossing and completed their last crossing after less time in training than horses in the massed training condition. Overall, the data suggest that compared with massed training, intra-session spaced training leads to a greater number of animals reaching task proficiency with faster task acquisition. This is particularly impressive given the relatively short training sessions of 8-min per training day.

The present data add to the prior report of ponies showing the spaced-training effect (Rubin et al. 1980), suggesting that the spacing effect generalizes to horses as well as to humans (Cepeda et al. 2006; Dunlosky et al. 2013), rats (Bello-Medina et al. 2013), mice (Aziz et al. 2014), dogs (Demant et al. 2011), pigs (Karas et al. 1962), honeybees (Menzel et al. 2001), and drosophila (San Martin et al. 2017). In addition to that theoretical point, the present data are important for applied settings because the task employed intra-session spacing. Spacing between real-world training sessions is often influenced by unavoidable constraints like equine injury, travel for performance, and the inconsistencies of human work and social schedules. Intra-session spacing accommodates this variability and can be applied anywhere from isolated training sessions to consistent, longitudinal training programs. Learning success may decrease frustration and increase positive interactions between horses and their trainers. Adding the intra-session spaced training method to trainers’ toolkits sets them up for more efficient training. Future studies with a variety of species should manipulate intra-session spacing to assess its generalization across species.

The design and method of the present study were chosen to maximize applied relevance while maintaining the experimental control needed to infer causation. Attributes of the design that support the applied contribution of this work given their face validity include: (1) using intra-session spacing rather than inter-session spacing where trainers have more control; (2) using an obstacle-crossing task that generally mimics a number of typical training scenarios, including creek crossings, trailer loading, entering a gate, navigating a jump, or passing by any frightening novel stimuli; and (3) training the horses with a rider on their back using typical training cues (rein and leg pressure). Indeed, implementing a rider who utilized standard negative reinforcement-based cues is more aligned with standard industry practices compared to other research tasks that focus on positive reinforcement (Hothersall and Nicol 2007; McCall 2007; Murphy and Arkins 2007). Concurrently, attributes of the design that enhanced internal control include: (1) utilizing *both* random selection of horses from the herd *and* random assignment of horses to spaced or mass training conditions, (2) attempting to elicit a single trained response (forward motion over the obstacle), and (3) controlling for seven factors, namely (a) the total amount of training time per session, (b) the total amount of resting time per session, (c) the time of day that training was done, (d) the isolation of the testing area from other horses, (e) similar numbers of horses run in the massed and spaced training conditions in the majority of training sessions, (f) the same hypothesis-naive rider/saddle equipment for all horses including the fact the rider did not ride the horses during normal work activities, and (g) the breakdown of the obstacle daily so that horses’ only exposure to it was during training. The present methodology, with high internal control and high external generalizability, holds promise for future experimental manipulation that still conforms to standard industry practices.

Moreover, the present study also extends what is known about the spacing effect to negative reinforcement. A literature search for studies that combined *spaced practice* or *spaced learning* or *spaced retrieval* with *negative reinforcement* yielded only one study (Beattie and Corr 2010). A second search for studies that combined *spaced training* with *negative reinforcement* yielded five more. Further examination of these studies revealed that two focused on punishment rather than negative reinforcement (Beattie and Corr 2010; Wimmer et al. 2018) and two examined positive rather than negative reinforcement (Amsel et al. 1971; Marx 1969). One study reported that spaced training was more effective than massed training with negative reinforcement in mice (Stern et al. 2020). Finally, one study included horses (McCall et al. 1993) trained on an avoidance (Pavlovian) conditioning task. McCall et al. (1993) concluded that moderate repetition of training (roughly 15 trials per session) maximized learning efficiency which they contrasted with intense activity in few sessions. In our view, the authors are arguing that spaced training is better than massed training, as are we. This brief review highlights that there is a gap in the literature to be filled with future work on spaced training and negative reinforcement. Notably Wimmer et al. (2018) highlight the need for more ecologically relevant research; the present study answers this call and inspires follow-up work such as the role of arousal in learning in spaced and massed conditions.

As with any study, there are limitations of the present work. Between-group designs always beg the question of sampling bias. Importantly, both the lack of difference in the training groups’ approach behavior in the first 2 min of training and the fact that neither group had a horse cross within the first 2 min reduce this concern. Naturalistic settings always reduce experimental control compared with laboratory settings. The present study included some variation in the time between training sessions and the number of horses that could be run in each condition on a given day (e.g., weather making it unsafe to continue training). Replication of this work in laboratory settings can better control for precise training timing than was possible in the present study. The field setting precluded us obtaining detailed data about the horses’ ages. We hope that future work that builds upon the present findings can explore whether variables such as horse age (and prior experience) affect data patterns in non-human animals including horses, although we note that older adult humans show a similar spacing effect to younger adult humans (e.g., Kornell et al. 2010).

Trained equids are often asked to respond to stimuli and perform in environments that are at odds with their evolutionary programming as prey animals (Christensen et al. 2010). Any measures taken to increase training efficiency have the potential to reduce stress and injury to both horse and horse handler. The demonstrated benefits of intra-session spaced training on equine learning performance justify additional research and hold promise for increased learning efficiency in all forms of equine training.

## Data Availability

The data are on the Open Science Framework at this link: https://osf.io/kx6gm/?view_only=e464745032ba4d408aa5ffd575bb4dad.
